# Acinetobacter variabilis represents a diverse species with novel regions associated with antibiotic resistance and surface polysaccharides

**DOI:** 10.1099/mgen.0.001643

**Published:** 2026-02-13

**Authors:** Lucy L. Patterson, Eva Hatje, Mohammad Katouli, Johanna J. Kenyon, Mehrad Hamidian

**Affiliations:** 1 Australian Institute for Microbiology and Infection, University of Technology Sydney, Ultimo, NSW, Australia; 2 Centre for Immunology and Infection Control, Faculty of Health, School of Biomedical Sciences, Queensland University of Technology, Brisbane, QLD, Australia; 3 Centre for Bioinnovation, and School of Science, Technology and Engineering, University of the Sunshine Coast, Maroochydore, QLD, Australia; 4 School of Pharmacy and Medical Sciences, Health Group, Griffith University, Parklands Drive, Gold Coast, Queensland, 4222, Australia; 5 Institute for Biomedicine and Glycomics, Griffith University, Parklands Drive, Gold Coast, Queensland, 4222, Australia

**Keywords:** *Acinetobacter variabilis*, antibiotic resistance, *bla*_NDM_, *bla*_OXA-23_, plasmid

## Abstract

*Acinetobacter variabilis* is an opportunistic pathogen found in both clinical and environmental settings with the potential to harbour and disseminate clinically significant antibiotic resistance genes. Here, we sequenced the complete genome of NGH-QLD-N1, an *A. variabilis* isolate recovered from a blood sample of a patient at Nambour General Hospital located on the Sunshine Coast region of Queensland, Australia. The assembly was used as a reference to assess the diversity in available *A. variabilis* genome sequences and investigate the genomic context of surface polysaccharide biosynthesis genes and antibiotic resistance determinants in relation to the priority pathogen, *Acinetobacter baumannii*. Phylogenetic analysis revealed substantial diversity in the species, with a distinct NGH-QLD-N1 subclade containing multiple strains with important resistance determinants. We identified a novel transposon related to the Tn*6022* family, designated Tn*6929*, that interrupted *comM* and identified multiple structural variants in *A. variabilis* genomes. Notably, some variants carried *bla*_NDM_ and *aphA6* in ISAba14-bounded regions, suggesting acquisition from diverse bacterial hosts. We also identified several integrative conjugative elements (ICEs) variants integrated into the chromosomal *thyA* gene, which differed in backbone structure and resistance gene content from our previously described *A. baumannii* ICE. The capsule and outer core loci for surface carbohydrate structures also had similar arrangements to those found in *A. baumannii*. This study provides a new understanding of the diversity of mobile genetic elements and ICEs carrying resistance genes, and surface polysaccharides associated with virulence, across the *Acinetobacter* genus.

**Impact Statement**

This study revealed extensive genomic diversity within the *Acinetobacter variabilis* species, including significant variation in surface polysaccharides, antibiotic resistance gene structures and mobile genetic elements associated with them. The presence of Tn*6929* and diverse integrative conjugative elements in *A. variabilis* highlights its potential to disseminate key resistance genes such as *bla*_OXA-23_, *bla*_OXA-58_ and *bla*_NDM_ to other species. By analysing the diversity and evolutionary potential of these mobile elements, this work provides the foundation for understanding the genomic context and mobility of resistance genes in *A. variabilis*, which is important for studying the emergence and evolution of multidrug resistance within *Acinetobacter* species and across the genus more broadly.

## Data Summary

The *Acinetobacter variabilis* NGH-QLD-N1 genome sequenced in this study is deposited in GenBank and available under the BioProject PRJNA1304695. All 64 publicly available *A. variabilis* genomes used in this study were downloaded from GenBank with their accession numbers listed in Table S1.

## Introduction

The global spread of multidrug-resistant (MDR) bacterial pathogens poses a significant threat to public health and has complicated the treatment and management of infectious diseases across healthcare settings [1,2]. Among these, members of the genus *Acinetobacter*, particularly *Acinetobacter baumannii*, have been extensively studied due to their high levels of antibiotic resistance and global dissemination [3,5]. As of July 2025, the *Acinetobacter* genus includes 119 species (https://lpsn.dsmz.de/genus/acinetobacter). While some have found their way into hospitals, causing serious healthcare-associated infections, the majority of species are commonly found in natural and non-clinical environments (e.g. soil, water, food production and domestic animals) [6]. Despite emerging reports of their role in human infections and their potential to carry clinically significant antibiotic resistance genes (ARGs) and virulence genes, non-*baumannii* species within the *Acinetobacter* genus remain relatively understudied.

Non-*baumannii* species have been suggested to serve as reservoirs for ARGs, increasing diversity and spread, particularly through mobile genetic elements. Recently, we showed that plasmids play an important role in the dissemination of ARGs across the *Acinetobacter* genus [7,8]. These plasmids have been implicated in the spread of carbapenemases, aminoglycoside-modifying enzymes and other significant resistance determinants. Characterizing the structure, content and phylogenetic context of plasmids within less-studied *Acinetobacter* species is therefore important to understand their contribution to the potential for interspecies ARG acquisition and spread.

In 2015, *Acinetobacter variabilis* sp. nov. (formerly known as genospecies 15 sensu Tjernberg and Ursing) was officially named as a novel species isolated from a variety of human clinical specimens and animals [9]. Since the identification of this species, few studies have reported *A. variabilis* strains recovered from a variety of clinical, environmental and animal samples in different countries in Asia, Europe and the USA [10,13] with no reports from Australia. The earliest reported isolate is the type strain, * A. variabilis* NIPH 2171, which had been recovered from the urine of a patient in Malmö, Sweden, in 1980–1981 (GenBank accession number APRS01000000) [14]. However, despite early isolation and numerous reports [10,13], analysis of genomic data for * A. variabilis* remains limited, with only 64 whole-genome sequences, and none from the Oceania region, currently available in NCBI databases (as of July 2025). There have also been a limited number of studies reporting the genetic context of antibiotic resistance determinants [10,12, 13], or that have investigated the broader diversity in virulence genes across the species, including for the capsular polysaccharide (CPS) and lipooligosaccharide (LOS).

*A. variabilis* isolate NGH-QLD-N1 was recovered in 2010 from a blood sample of a patient at the Nambour General Hospital (NGH) on the Sunshine Coast region of Queensland, Australia. Here, we sequenced the complete genome of NGH-QLD-N1 using short- and long-read sequencing data and confirmed the isolate as a member of the *A. variabilis* species. We examined its genomic features and phylogenetic relationship with other *A. variabilis* genomes and analysed the genomic context of ARGs and loci for major virulence determinants, including the CPS and LOS. Our findings show that transposons related to the Tn*6022* family, which are frequently found in *A. baumannii*, integrative conjugative elements (ICEs) and plasmids found in *A. variabilis* may serve as a reservoir and play a significant role in driving antimicrobial resistance spread across the genus.

## Methods

### Bacterial isolate and antibiotic resistance profile

NGH-QLD-N1 was recovered in a blood sample of a patient at NGH, Queensland, Australia, in 2010. The antibiotic resistance profile of NGH-QLD-N1 was determined against 23 antibiotics (shown in Table S1, available in the online Supplementary Material) as we described previously [15] using the standard Calibrated Dichotomous Sensitivity disc diffusion method [16].

### Whole-genome sequencing and assembly

Whole-cell DNA was isolated from a single colony of *A. variabilis* NGH-QLD-N1 cultured in Luria–Bertani medium at 37 °C overnight using the PureLink^™^ Genomic DNA mini kit (Thermo Fisher Scientific). Concentration was measured using the Invitrogen Qubit system and purity by spectrophotometry (NanoDrop, Thermo Fisher Scientific). The whole-genome sequence was obtained via hybrid sequencing: Illumina MiSeq at the Australian Genome Research Facility, Australia, and Oxford Nanopore Technology at Plasmidsaurus, USA. Raw read quality was assessed with *FastQC* [17], and adapter trimming was performed using *Btrim* v0.2.0 [18]. *De novo* assembly of the complete genome was completed using *Autocycler* v0.1.2 (https://github.com/rrwick/Autocycler/tree/v0.2.0), followed by polishing with the Illumina data using *Polypolish* (https://github.com/rrwick/polypolish). The complete genome assembly consisted of a 3,230,105 bp chromosome and 6 plasmids ranging in size from 7.8 kb to over 210 kb. The final assembly was submitted to NCBI and is available under BioProject PRJNA224116 and BioSample SAMN50566246, and accession number JBQTGN000000000.

### Collation and annotation of available *A. variabilis* genome sequences

As of July 2025, a total of 64 genome assemblies were available in NCBI for the *A. variabilis* taxon (ID: 70346). Genome quality, including completeness and contamination metrics, was evaluated using *CheckM* v1.2.3 [19] for all genomes analysed, including NGH-QLD-N1 sequenced in this study and those retrieved from NCBI. This led to 13 genomes being removed from further analyses due to a *CheckM* completeness score of zero. Hence, a total of 52 genomes, including *A. variabilis* NGH-QLD-N1, were analysed in this study (accession numbers listed in Table S1). Whole-chromosome sequence alignments were constructed using *Proksee* [20] or *pyGenomeViz* (https://github.com/moshi4/pyGenomeViz) using the blast workflow with default parameters.

ARGs were detected using the *AMRFinderPlus* v.4.0 program [21]. Plasmid replicon types (*rep*/Rep) were identified using the *Acinetobacter* plasmid typing scheme [22]. Insertion sequences were identified using *ISFinder* [23] and Standalone blast v2.16.0 [24]. Prophage elements were detected using *PHASTER* (PHAge Search Tool Enhanced Sequence Translation; https://phaster.ca/). Virulence genes, including CPS (KL) and LOS outer core (OCL) biosynthesis loci, were manually identified by searching for homologues of *A. baumannii* proteins using BLASTp and were annotated consistent with the nomenclature system described for *A. baumannii* [25]. Protein-coding sequences were functionally annotated via *UniProt* [26] and *InterPro* [27]. Average nucleotide identities (ANIs) of the complete genomes were calculated using *FastANI* v1.34 (available at https://github.com/ParBLiSS/FastANI). Genomic regions of interest were visualized using *SnapGene*^®^ v6.0.5 or *Clinker* v0.0.28 (available at https://github.com/gamcil/clinker) and then annotated in *Illustrator*^®^ v26.2.1.

### Phylogenetic analysis and pangenome reconstruction

Maximum-likelihood phylogenetic (MLP) trees were constructed (using the NIPH 2171 genome as a reference) by aligning genome sequences using *Panaroo* [28] and analysed with *IQ-TREE* v2.3.6. The high-quality core-genome SNPs were identified and exported for further refinement. The final MLP tree was inferred from the alignment of substitution mutations rooted using an *A. baumannii* outgroup (strain ATCC 17978; GenBank no. CP012004). The final MLP tree was visualized using *FigTree* v1.4.4. Pangenome analysis was performed using *Panaroo* [28] in ‘strict mode’ with the core defined as genes present in 100% of genomes, the soft core as >95% and the shell genome as 15–95%. Additionally, antimicrobial resistance genes were plotted against the recombination-free tree using the plotTree v1.0 (https://github.com/katholt/plotTree) and *ggplot2* [29] v3.5.1 packages in *R*.

## Results and discussion

### Resistance profile, genomic features of NGH-QLD-N1 and *A. variabilis* genomes

**Fig. 1.**
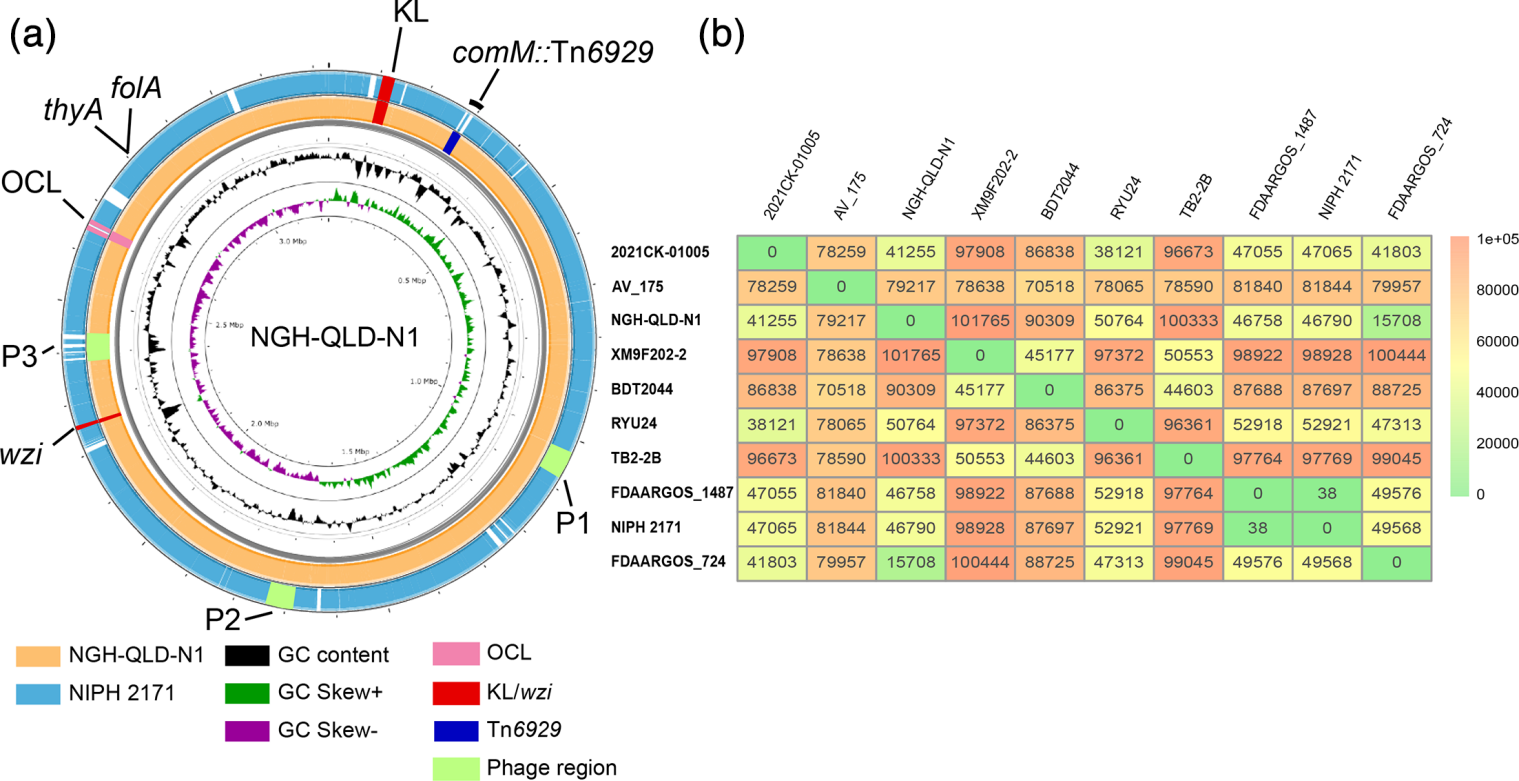
(a) *Acinetobacter variabilis* NGH-QLD-N1 genome, showing BLAST comparison to *A. variabilis* NIPH 2171 and the location of the KL/*wzi* and OCL loci, as well as Tn*6929* and phage regions. (b) Average nucleotide identity of nine complete and one near complete *A. variabilis* genomes. The number of single nucleotide differences are shown and also indicated by the coloured scale.

A high-quality hybrid genome assembly (Illumina and Oxford Nanopore) was obtained for NGH-QLD-N1, an isolate recovered from a blood infection at NGH in Queensland, Australia. The complete genome sequence was found to be a total of 3,517,317 bp with an average G+C content of 42.19 %, which included a 3,230,105 bp chromosome (Fig. 1a; NCBI accession number CM126357.1) and 6 plasmids ranging in size from 7.8 kb to over 210 kb (Table 1). NGH-QLD-N1 lacked the intrinsic *ampC* and *oxaAb* genes, which are characteristic for *A. baumannii*, suggesting that it is non-*baumannii*. To determine the species, we compared the DNA sequences of several chromosomal genes, including the *recA* and the 16S rRNA genes, with those of other known *Acinetobacter* species and found 100% identity with *A. variabilis* genomes. To confirm the species, phylogenetic analysis was performed with reference genomes of all *Acinetobacter* species (over 90 species), showing NGH-QLD-N1 clustering with the representative *A. variabilis* genome (Fig. S1). This was further validated by ANI analysis, which showed 95.23% identity across the genome.

**Table 1.** Properties of *A. variabilis* genomes studied here

Strain/plasmid	Year	Country	Isolation site	Size (bp)	Resistance genes	Plasmid Rep type	*comM* GI	Accession no.
**NIPH 2171**	1981	Sweden	Urine	Draft	–		*comM* intact	APRS00000000
**NGH-QLD-N1**	**2010**	**Australia**	**blood**	3,230,105	–	–	**Tn*6929*::ISAcsp13**	JBQTGN010000001
p1NGH-QLD-N1				7,839	–	R3-T18		JBQTGN010000002
p2NGH-QLD-N1				7,851	–	R3-T77		JBQTGN010000003
p3NGH-QLD-N1				10,584	–	R3-T62		JBQTGN010000004
p4NGH-QLD-N1				12,401	–	–		JBQTGN010000005
p5NGH-QLD-N1				37,499	–	–		JBQTGN010000006
p6NGH-QLD-N1				210,678	–	R3-T45		JBQTGN010000007
FDAARGOS_724*	nk†	USA	Unknown	3,235,776	–	–	Tn*6929*::ISAlw34-like ::ISAba12::ISAba22	CP054908
unnamed1				5,829	–	–		CP054904
unnamed2				214,175	–	R3-T45		CP054907
unnamed3				5,819	–	R3-T85		CP054906
unnamed4				6693	–	R3-T83		CP054905
**2021CK-01005**	2021	USA	Wound/abscess	3,174,744	*bla*_OXA-23_, *sul2*, *floR*	–	Tn*6929*::ISAba125::ISAba16	CP104651
unnamed1				185,297	–	R3-T45		CP104652
unnamed2				123,124	*bla*_OXA-58_, *bla*_NDM-1_, *bla*_MBL_, *mph-msr*(E), *strAB*, *aphA6*, *aacC2d*	R3-T28		CP104653
unnamed3				6,631	–	R3-T177		CP104654
unnamed4				4,872	–	R3-T26		CP104655
unnamed5				4,326	–	R3-T134		CP104656
unnamed6				2,621	–	R3-T96		CP104657
unnamed7				2,366	–	R1-T23		CP104658
**RYU24**	2012	Japan	Faecal swab	3,198,423	*strAB*, *sul2*, *dfrA* (novel)	–	Tn*6929*	AP024524
pRYU24				68,069	*mph-msr*(E), *armA*, *sul1*, *bla*_OXA-420_, *bla*_CARB-2_, *bla*_MBL_, *bla*_NDM_, *aphA6*	R3-T60		LC591943
**AV_175**	2016	Pakistan	Clinical	3,252,197	*bla*_MBL_, *bla*_NDM_, *sul2*, *floRstrAB*, *aphA6*, *folA*	–	Tn*6929*::TnAba14::Aba25::Aba27	CP078027
pAV_175-2				196,965	–	R3-T90		CP078028
pAV_175-3				19,844	–	R3-T144		CP078029
pAV_175-4				19,843	*tet*39, *mph-msr*(E)	R3-T10		CP078030
pAV_175-5				15,472	–	R3-T29		CP078031
pAV_175-6				4,226	–	RP-T11		CP078032
**TB2-2B**	2022	China	Soil	3,171,395	*sul2*, *strAB*, *floR*	–	*comM* intact	CP130328
pTB2-2B-1				141,842	–	R3-T90		CP130326
pTB2-2B-tetX				83,648	*tet(X5*)(2×), *tet(X3*)(2×), *sul2*, *aphA1*(2×), *floR* (3×)	R3-T63		CP130330
pTB2-2B-3				18,235	–	R3-T116		CP130329
pTB2-2B-4				11,798	–	R3-T12		CP130324
pTB2-2B-5				9,336	–	R3-T176		CP130325
pTB2-2B-6				7,661	*mph-msr*(E)	R3-T60		CP130327
**FDAARGOS_1487**	nk†	Germany	Unknown	3,211,351	–	–	*comM* intact	CP083658
unnamed1				158,619	–	R3-T90		CP083659
unnamed2				89,812	–	R3-T80		CP083660
unnamed3				13,782	–	R3-T29		CP083661
unnamed4				11,089	–	R3-T11		CP083662
**XM9F202-2**	2020	China	Chicken faeces	3,171,535	*dfrA40*, *tet(X15)*	–	*comM* intact	CP060811
pXM9F202-2-2k				2,301	–	R1-T24		CP060816
pXM9F202-2-13k				13,130	–	R3-T77		CP060815
pXM9F202-2-17k				17,946	–	R3-T116		CP060814
pXM9F202-2tetX-90k				90,430	*inu(G)*, *strAB*, *sul2*, *tet(X3)*, *dfrA1*, *sat2*, *aadA1*, *aphA1*, *aacC2e*, *floR*	R3-T63		CP060813
pXM9F202-2-186k				186,242	*tet(M)*, *sul2*, *aphA1*, *strAB*, *aacC2d*, *mph-msr*(E), *floR*	R3-T90		CP060812
**BDT2044**	2020	China	Pig faeces	3,345,806	*strAB*, *tet(X3)* (7×)	–	*comM* intact	CP094246
pBDT2044-1				10,546	–	R3-T196		CP094247
pBDT2044-2				17,439	*floR*	–		CP094248
pBDT2044-3				18,754	*tet39* (2×)	R3-T76		CP094249
pBDT2044-4				20.030	–	R3-T84		CP094250
pBDT2044-5				36,028	–	–		CP094251
pBDT2044-6				46,646	*bla*_OXA-23_	–		CP094252
pBDT2044-7				55,901	*aphA1*, *floR*	R3-T20		CP094253

*Reported as *Acinetobacter *sp.

†nk, not known.

The general features of NGH-QLD-N1 were compared with eight other complete *A. variabilis* genomes identified in the NCBI database and one near-complete draft genome (contigs *n*=23) for the *A. variabilis* type strain NIPH 2171 (Table 1). Like NGH-QLD-N1, the average chromosome size of these genomes ranged from 3.1 Mb to just over 3.3 Mb, and their G+C content ranged from 42.04 to 42.38%, indicating that the average G+C content for *A. variabilis* is just over 42%. In comparison to *A. baumannii*, the average chromosome size for *A. variabilis* is significantly smaller (typically 4 Mb in *A. baumannii*), while the G+C content is higher (e.g. 39.01% for *A. baumannii* ATCC 17978 and 39.17% for *A. baumannii* ATCC 19606; determined here). Pairwise genome comparisons among the 10 available complete genomes (Fig. 1b) revealed between 38 and 101,765 single-nucleotide differences (SNDs), corresponding to roughly 99.99% down to ~97.1% genome-wide nucleotide identity across a ~3.5 Mb genome. While based on a limited dataset, this range of divergence is comparable to levels of within-species genomic heterogeneity reported for other *Acinetobacter* species, including *A. baumannii* [30,31], and is consistent with the genus-level pattern of an open pangenome and high genomic plasticity [32]. The observed SND range is indicative of genomic heterogeneity among the currently sampled members of this species. However, as additional genomes become available, the extent and structure of this diversity can be further evaluated and refined.

The NGH-QLD-N1 chromosome was therefore aligned with the earliest recovered isolate, NIPH 2171 (Fig. 1a), and several regions of difference were identified. One of these was found to be a prophage region (P3 in Fig. 1a), whereas others indicated differences at *comM*, a common location disrupted by antibiotic resistance islands in *A. baumannii* [3,4, 33], and a locus predicting synthesis of a surface polysaccharide (see below). NGH-QLD-N1 was also found to include several IS, including six copies of ISAcsp13, four copies of ISAba25 and many novel IS, including one distantly related to ISAba59 (IS5 family) with 87% DNA identity and a second novel IS distantly related to ISAlw33 (IS30 family), also with 87% DNA identity. The latter two IS (related to ISAba59 and ISAlw33) were also present in NIPH 2171.

To further identify whether the regions of difference were associated with the locations of resistance determinants, the NGH-QLD-N1 genome was searched for ARGs. However, none were identified. The isolate was therefore tested against a panel of 23 antibiotics and found to be susceptible to all antibiotics tested except for trimethoprim (Table S1). Further analysis of the genome revealed that NGH-QLD-N1 carried a single chromosomal resistance gene (locus tag ACTNFC_13365) encoding a FolA homologue (97.65% aa identity to DfrA40) that would account for resistance to trimethoprim [34]. This gene was also identified in the NIPH 2171 chromosome (Fig. 1a) and the other complete genomes, suggesting that the species may be intrinsically resistant to trimethoprim.

### *A. variabilis* species has a diverse pangenome

**Fig. 2.**
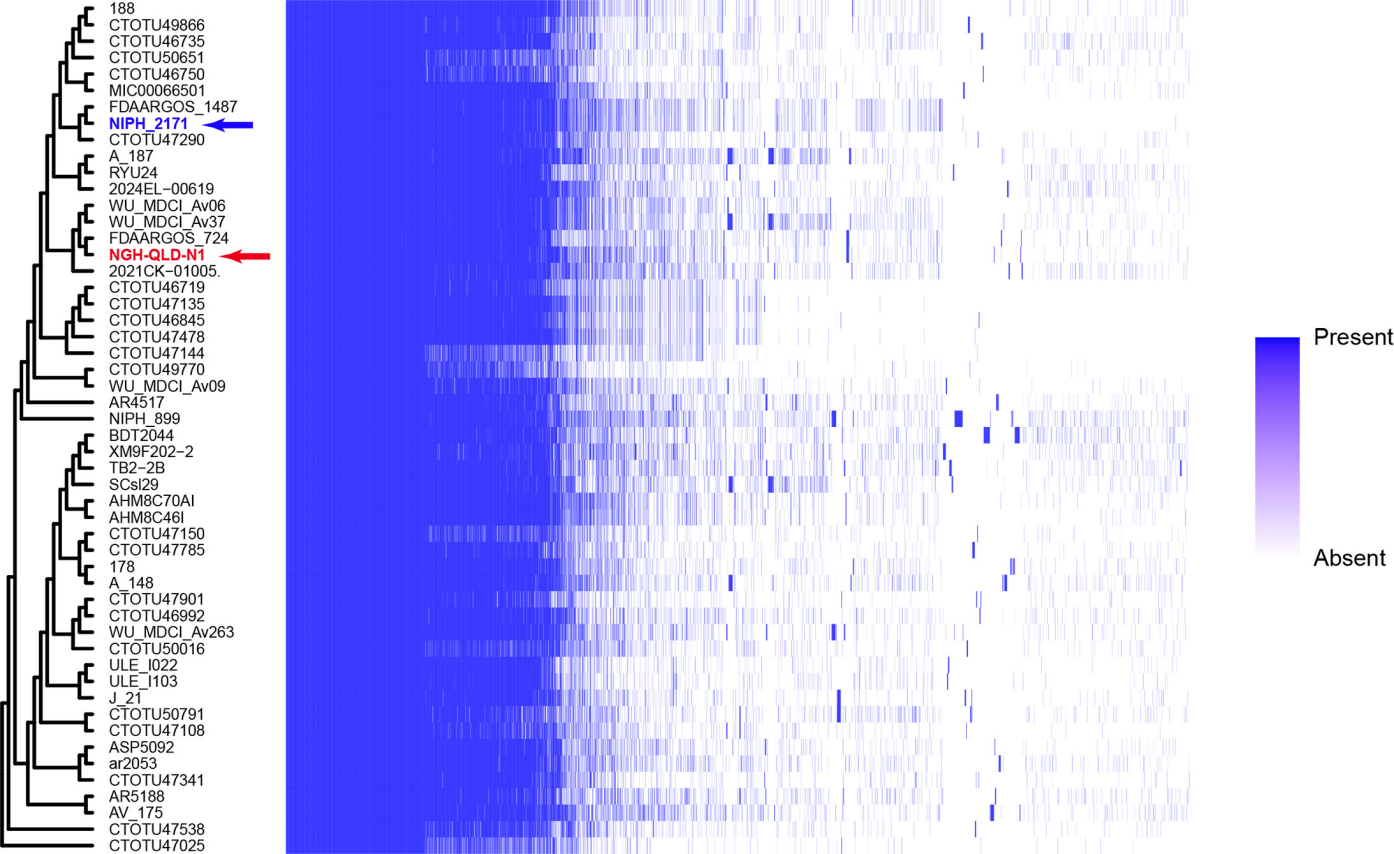
Phylogenetic tree and the pangenome heatmap of 52 *Acinetobacter variabilis* genome sequences. The pangenome contains 8, 410 unique genes with gene presence/absence indicated by the blue scale shown.

A total of 64 genome assemblies, including NIPH2171 and the 8 complete genomes, were identified in NCBI under the *A. variabilis* taxon (ID: 70346). Completeness and contamination of all 64 genomes were assessed using *CheckM*, and 13 draft assemblies did not pass our quality metrics and were therefore removed from the dataset. The final pool of 52 genomes (including NGH-QLD-N1) represented diverse isolates recovered from a range of clinical and non-clinical samples (i.e. soil, water, food and animals) from several countries across Asia, Europe and the USA. However, none from NCBI were found to originate from South America or Australia. Hence, NGH-QLD-N1 represents the first *A. variabilis* genome to be reported from the Oceania region. Pangenome analysis of the *A. variabilis* set identified a total of 8,410 unique genes, with a relatively small core genome of just 15.4% (1,294 genes across 52 genomes) (Fig. 2). The accessory genome was extensive, comprising 905 soft core genes, 1,329 shell genes and 4,882 cloud genes (Fig. 2). Our dataset included a diverse collection of *A. variabilis* genomes, likely representing multiple sequence types, comparable to previous studies of diverse *A. baumannii* genomes, which reported core genomes of ~13% [35,36]. The genomic diversity identified across the *A. variabilis* population supports the original phenotypic heterogeneity observations, which inspired the species name ‘variabilis’, reflecting its variable nature [9].

### Phylogenetic analysis of *A. variabilis* genomes

**Fig. 3.**
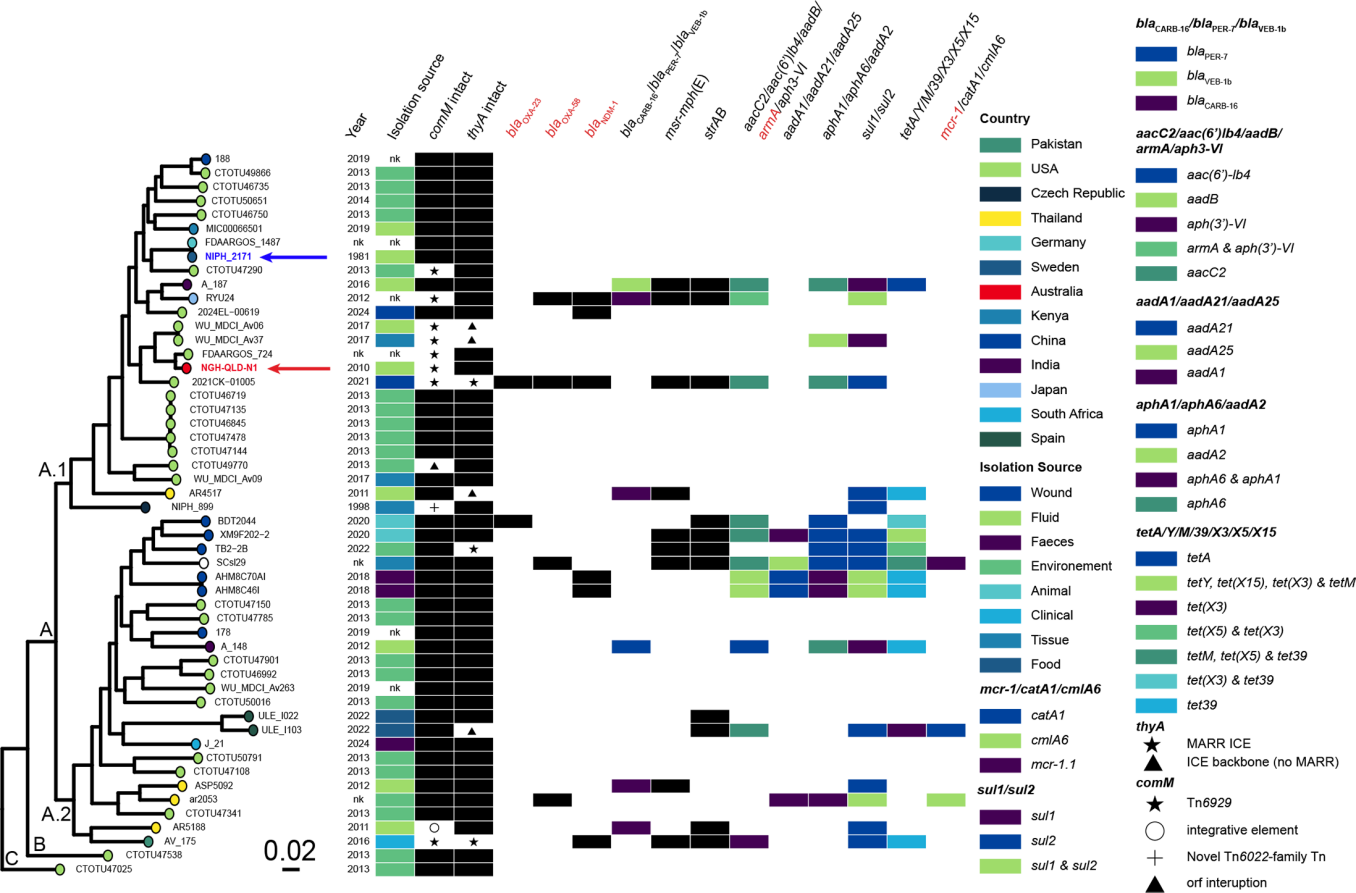
Maximum likelihood phylogenetic tree of *Acinetobacter variabilis* (n=52) genome sequences. Next to each *A. variabilis strain* the year, country and source of isolation are shown. Presence of antimicrobial resistance genes are also indicated, by the colour scheme shown.

To assess the population structure of the species, an MLP tree was constructed using NGH-QLD-N1 and the 51 publicly available *A. variabilis* genomes. The phylogeny (Fig. 3) revealed three major clades, named A, B and C, with clade A comprising the majority of isolates in two subclades (A.1 and A.2). However, clades B and C each contain a single genome and were regarded as provisional lineages as their distinct phylogenetic placement may reflect under-sampled population structure rather than well-defined clades. Both NGH-QLD-N1 and the earliest isolate, NIPH 2171, were found in A.1 (highlighted in Fig. 3), which is hereafter referred to as the NGH-QLD-N1 subclade. The eight other complete genomes were found across multiple clades in the phylogeny. Genomes in clade A originated from diverse geographical regions, including the USA, Kenya, South Africa, Pakistan and several European and Asian countries, with all but two (NIPH 2171 from 1981 and NIPH 899 from 1998) recovered after 2010 (Fig. 3).

Each genome was assessed for known ARGs, and this information was overlaid on the phylogeny. While ARGs were not detected in NGH-QLD-N1 and several other isolates predominately within the NGH-QLD-N1 subclade, 21 genomes from diverse geographical regions contained at least 1 resistance gene. Except for a few related genomes (e.g. see Fig. 3), these genomes generally belonged to unrelated phylogenetic sub-lineages and were distributed across the phylogenetic tree. However, despite the relatively low abundance of resistance genes in *A. variabilis* genomes in general, several clinically significant carbapenemase genes, including *bla*_OXA-23_, *bla*_OXA-58_ and *bla*_NDM_, the 16S rRNA methyltransferase gene *armA*, conferring high-level resistance to all aminoglycoside antibiotics, and the *mcr-*1 colistin resistance genes were detected. Several strains from diverse geographical regions, including China, Thailand, Pakistan and the US, also contained *tetA*(A), *tetY*, *tetM*, *tet39*, *tetX3*, *tetX5* and *tetX15* tetracycline resistance genes (Fig. 3). Overall, with the exception of five strains from China that were grouped together (XM9F202−2, BDT2044, TB2−2B, AHM8C46I and AHM8C70AI; Fig. 3), the remaining genomes with multiple resistance genes were distributed across the phylogenetic tree without a distinct geographical or temporal pattern.

Within the NGH-QLD-N1 subclade, two closely related strains, RYU24 (Japan) and 2021CK−01005 (USA), along with two more distantly related genomes, AV_175 (Pakistan) and A_187 (China), contained multiple resistance genes, including those for carbapenems, aminoglycosides, sulphonamides and tetracyclines. Notably, RYU24 carried two carbapenemase genes, *bla*_OXA-58_ and *bla*_NDM_, while 2021CK−01005 carried three, with the addition of *bla*_OXA-23_. Eight genomes outside the NGH-QLD-N1 subclade also exhibited a similar profile, containing several clinically important resistance genes (Fig. 3).

*A. variabilis* genomes were further assessed for common virulence determinants in *A. baumannii* [37], and the results are summarized in Table 2. Most isolates encoded the Blc and SmpA outer membrane proteins, though OmpA homologues were found in less than half the genome pool. Very few isolates carried genes predicting pilin or siderophore biogenesis. However, almost all isolates encoded proteins associated with CPS biosynthesis and assembly [25], as well as a PglL homologue required for O-glycosylation of proteins with CPS glycan units [38]. A homologue of the WaaL ligase required for the attachment of an O-antigen polysaccharide to form LPS could not be detected, suggesting that, like *A. baumannii*, *A. variabilis* does not produce LPS with O-antigen but rather LOS consisting of the lipid A-core oligosaccharide components only [25,39]. Consistent with this hypothesis, the majority of genomes encoded a homologue of Wzi required for mediating CPS assembly on the cell surface [40]. As for *A. baumannii*, the gene coding for this protein is located far from the CPS biosynthesis K locus (Fig. 1a) and, in * A. variabilis* genomes, is found between a lysine--tRNA ligase and tryptophan--tRNA ligase gene. An additional copy of *wzi* was also identified in some genomes (see below).

**Table 2.** Summary of virulence genes found in *A. variabilis* genomes

Gene	*A. baumannii* protein accession	Gene present (no. genomes)*			
		NGH_QLD_N1	NIPH 2171	Complete genomes (*n*=8)	Draft genomes (*n*=42)
**Outer membrane proteins**					
*blc*	AKA30801.1	+	+	+ (8)	+ (38)
*ompA*	AKA32295.1	−	−	+ (1)	+ (18)
*smpA*	AKA32747.1	+	+	+ (8)	+ (40)
**Biofilm formation**					
*csuA*	AKA31228.1	−	−	−	+ (3)
*csuB*	AKA31229.1	−	−	−	+ (3)
*csuC*	AKA31230.1	−	−	−	+ (4)
*pgaA*	AKA31295.1	−	−	−	−
*pgaB*	AKA31296.1	−	−	−	−
*pgaC*	AKA31297.1	−	−	−	−
*pgaD*	AKA31298.1	−	−	−	−
*bap*	AKA30645.1	−	−	−	−
*blp*	AKA30673.1	−	−	−	+ (32)
**Capsule biosynthesis**					
*wzc*	AKA33503.1	+	+	+ (8)	+ (38)
*galU*	AKA33489.1	+	+	+ (8)	+ (37)
*wzi*	AKA32612.1	+	+	+ (8)	+ (40)
**Lipopolysaccharide biosynthesis**					
*waaL*	(AAC69682.1)†	−	−	−	−
**Protein and pilin glycosylation**					
*pglL*	AKA30091.1	+	+	+ (8)	+ (39)
*tfpO*	WP_000914629.1	−	−	−	−
**Siderophore**					
*rhbC_3*	AKA31917.1	−	+	−	−
*lucD*	AKA31916.1	−	+	−	−
*emrB*	AKA31915.1	−	−	−	−
*rhbC_2*	AKA31914.1	−	−	−	−
*rhbC_1*	AKA31913.1	−	−	−	−
2Fe-2S	AKA31912.1	−	−	−	−
*rraA*	AKA31911.1	−	−	−	−
*bauA*	AKA30928.1	−	−	−	−
**Regulatory proteins**					
*bmfR*	AKA32888.1	+	+	+ (8)	+ (40)
*bmfS*	AKA32887.1	+	+	+ (8)	+ (38)
*gigA*	AKA32956.1	+	+	+ (8)	+ (39)
*gacA/S*	AKA33198.1	+	+	+ (8)	+ (40)

*Gene presence based on >85% coverage of encoded protein with >35% amino acid identity.

†*A. baumannii* does not produce WaaL*.* Accession is for a protein from *Escherichia coli*.

 As resistance and virulence genes are often found on plasmids or in genomic islands that can be highly fragmented in bacterial genomes, analysis of their genetic context was completed using the NGH-QLD-N1, NIPH 2171 and eight other complete genomes (Table 1).

### Plasmids

Similar to NGH-QLD-N1, most of the complete genomes contained at least four plasmids (Table 1). Interestingly, several plasmids were found to lack the R1, R3 or RP plasmid Rep types, which is also common in *A. baumannii* [7,8, 22]. However, variants of the R3-type (Rep_3) plasmids were among the most widespread. Among these R3-type Rep variants, R3-T80, T83, T85, T90, T116, T134, T173, T176 and T196 have not been detected in *A. baumannii*, while others, such as R3-T144 and T177, have been reported only in *A. variabilis* [7]. In contrast, variants such as R3-T11, T12, T20, T23, T29, T45, T60, T76 and T84 were found in multiple *A. variabilis* complete genomes (Table 1) [7]. In addition, we found several plasmids lacking R1, R2 or RP *rep*/Rep, but belonging to plasmid families we recently defined (e.g. the pA207-3 type and its variants) [7]. Among all complete plasmids, only pBDT2044-7 was predicted to be potentially conjugative, as it appeared to encode a complete set of conjugative transfer functions belonging to the MPF^T^ [41] system (locus IDs MOV98_17755–MOV98_17800 in GenBank accession CP094253).

Plasmids identified in 2021CK-01005, RYU24 and BDT2044 genomes carry several important ARGs, including the carbapenem resistance genes *bla*_OXA-58_, *bla*_OXA-23_ and *bla*_NDM_, as well as *armA* and the amikacin resistance gene, *aphA6* (Table 1). For plasmid pBDT2044-6, the *bla*_OXA-23_ carbapenem resistance gene was located in the Tn*2008* transposon, which is one of the common transposons that disseminate this gene in *Acinetobacter* [3,4]. For the MDR isolate RYU24, the genetic context of *bla*_NDM_, *bla*_OXA-420_ and *armA* genes in the 68, 069 bp plasmid, pRYU24, has been described previously [13], and each was reported to be surrounded by complete or remnant IS elements. Here, we identified that the *bla*_NDM_ gene was in a variant of Tn*125* (a transposon known to mediate the spread of *bla*_NDM_) with an IS*91* interrupting the *groL* gene, and we detected a remnant of *aphA6* interrupted by an IS*26* (immediately upstream). The archetypal form of Tn*125* carrying *bla*_NDM_ and *ble*_MBL_ was instead identified in the unnamed2 plasmid from 2021CK-01005. However, in this plasmid, *aphA6* was located immediately downstream of Tn*125* and was bounded by ISAba125 elements. The *bla*_OXA-58_ gene was located ~2–3 kb upstream of Tn*125*. Overall, in most complete *A. variabilis* genomes, plasmids appear to play an important role in carrying ARGs, as most ARGs were plasmid-borne rather than chromosomal.

### Variants of Tn*6929*, a novel transposon related to Tn*6022*-family transposons, carry the *bla*_NDM_ carbapenem resistance gene

**Fig. 4.**
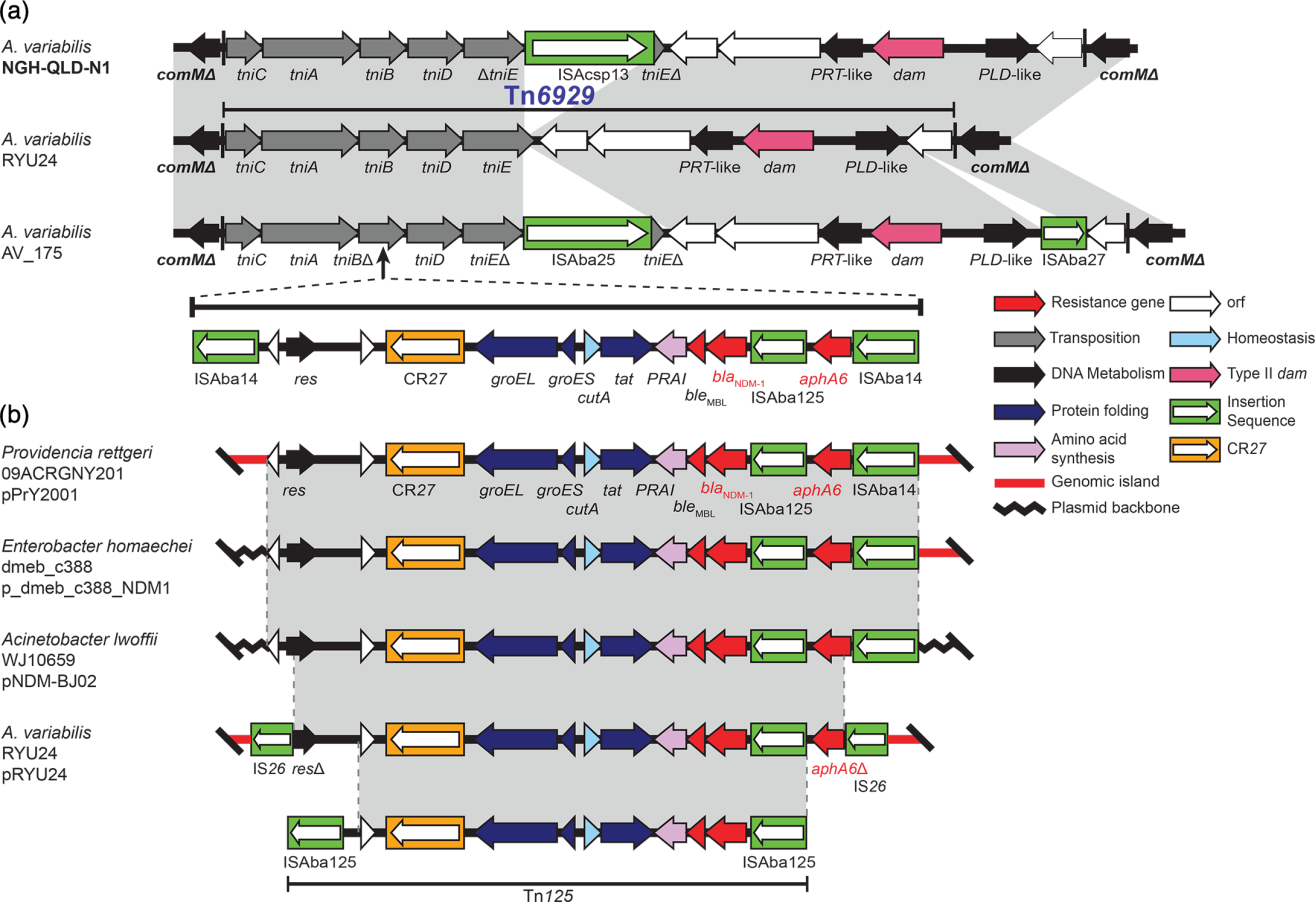
Genetic structure of a novel transposon in *A. variabilis***.** (a) Variants of the novel Tn*6929* in three *Acinetobacter variabilis* genomes, including the resistance region containing the ***bla***_NDM_ carbapenemase gene and the *aphA6* amikacin resistance gene in AV_175. (b) Comparison of the genetic structure of the AV_175 resistance region identified in closely related genera. Arrows indicate orientation and genes are coloured by their function. Open boxes coloured green show insertion sequences with their transposase gene indicated inside the boxes. Colour scheme is shown.

In *A. baumannii* strains belonging to major global clones, the chromosomal *comM* gene is often disrupted by large antibiotic resistance islands (AbaR-type in ST1, AbGRI-type in ST2 and AbaR4 in various other STs), which comprise transposon backbones (e.g. Tn*6019* and Tn*6022*, known as class III transposons) and a complex resistance region [33]. Analysis of the *comM* gene in NGH-QLD-N1 revealed disruption by a 17,051 bp element (bases 279,811–296,861 in GenBank accession no. JBQTGN010000001) showing characteristics like Tn*6019* and Tn*6022*, and other transposons belonging to this family [33]. This region (Fig. 4a) was found to include genes that encode a set of transposition functions (TniCABDE) related to those found in Tn*6019* and Tn*6022*. It was also found to be flanked by 26 bp inverted repeats (IRl: TGTCATATACTATAATAAAAGCTAGT and IRr: ACTAGCTTTTATTTTTGTAAATGACA), generating 5 bp (GCCAC) target site duplications (TSDs), and inserts precisely at the same location in the *comM* gene where variants of Tn*6019* and Tn*6022* and other large transposons belonging to this family (i.e. AbaR-type and AbGRI islands) are found. The *tni* module is more closely related to those encoded by Tn*6022* [42], Tn*6021* [33] and Tn*6173* [33], with 56–75% aa identities (Table 3), than to those of Tn*6019* [33] with no significant matches. However, the *tni* module shares other characteristics (i.e. IRs and TSDs) with all other known class III transposons.

**Table 3.** Amino acid identities of TniCABDE and orf4 (encoded by Tn*6019*, Tn*6022*, Tn*6021* and Tn*6173*) compared to those encoded by Tn*6929*

Tn	TniC	TniA	TniB	TniD	TniE	orf4
Tn*6019*	–*	–	–	–	–	–
Tn*6022*	71.41%	69.78%	71.37%	67.38%	68.58%	27.56%
Tn*6021*	75.93%	72.28%	71.37%	67.38%	66.41%	27.56%
Tn*6173*	67.53%	66.61%	73.03%	57.18%	56.01%	26.30%

*No significant identity.

In NGH-QLD-N1, the backbone of this Tn is interrupted by an ISAcp13. However, an intact version without this interruption was identified in the complete genome of isolate RYU24 (bases 265,584–280,057; GenBank accession no. AP024524). Hence, this intact version was named Tn*6929.* The sequence was found to encode a putative methylase (annotated as Type II *dam* gene in Fig. 4) that belongs to the IPR000241 protein family (Table 1). The *comM* gene was found intact in NIPH2171, BDT2044, XM9F202-2, FDAARGOS_1487 and TB2-2B. However, additional variants of Tn*6929* were identified in three of the other complete genomes (Table 1). For example, FDAARGOS_724 included a version interrupted by ISAlw34, ISAba12 and ISAba22, and 2021CK-01005 contained another version interrupted by ISAba125 and ISAba16.

Notably, *A. variabilis* AV_175 (GenBank no. CP078028) includes a much larger version of Tn*6929*, containing a 14,275 bp resistance region that carries the *bla*_NDM_ carbapenemase gene and the *aphA6* amikacin resistance gene (Fig. 4a). This region is bounded by two copies of ISAba14 and flanked by 3 bp TSDs characteristic of ISAba14 insertion. While the internal segment of the *bla*_NDM_-*aphA6* region was identical to Tn*125*, it was different to the forms found in pRYU24 and the 2021CK-01005 unnamed2 plasmid (described above). Hence, it appears that the 14 kb ISAba14-bounded resistance region was acquired from another variant of this region, also associated with ISAba14. Supporting this hypothesis, we identified several closely related regions in distantly related genera (Fig. 4b), including the pPrY2001 plasmid (*Providencia rettgeri* strain 09ACRGNY201; GenBank no. KF295828), the p_dmeb_c388_NDM1 plasmid (*Enterobacter hormaechei* strain dmeb_c388; GenBank no. CP095673) and the pNDM-BJ02 plasmid (*Acinetobacter lwoffii* strain WJ10659; GenBank no. JQ060896). These findings highlight the potential for ISAba14-associated resistance regions to disseminate across diverse bacterial hosts, posing a risk for interspecies transfer of the most clinically significant carbapenem resistance gene, *bla*_NDM_. It also demonstrates the potential for Tn*6929* to spread to other *Acinetobacter* species, including *A. baumannii*, given the proven ability of class III transposons (i.e. Tn*6022* and Tn*6019*) [4,33] to capture and mobilize resistance genes within globally disseminated, high-risk clones.

Amongst the draft *A. variabilis* genomes, 37 included an intact *comM* gene (Fig. 3), and 8 contained a variant of Tn*6929*. Variants of Tn*6929* were identified in distinct branches of the phylogenetic tree, indicating that these acquisitions occurred independently on multiple occasions. A single genome, NIPH 899, included a novel cryptic transposon (29,543 bp Tn, located at bases 262,001–291,543 in GenBank no. APPE01000087) belonging to the Tn*6019*–Tn*6022* family. This region encodes several proteins involved in various metabolic pathways and many hypothetical proteins. The *comM* gene was also interrupted by a novel ICE in AR5188 (GenBank no. JAZHCM000000000) and CTOTU47290 (GenBank no. DAISMA000000000). These findings highlight the role of transposons related to Tn*6019*, Tn*6022* and Tn*6929* to spread a wide range of genetic material and the *comM* gene as a hotspot to capture these elements in different *Acinetobacter* species, including *A. baumannii* and *A. variabilis*.

### ICEs as potential hotspots for ARG acquisition in *A. variabilis*

**Fig. 5.**
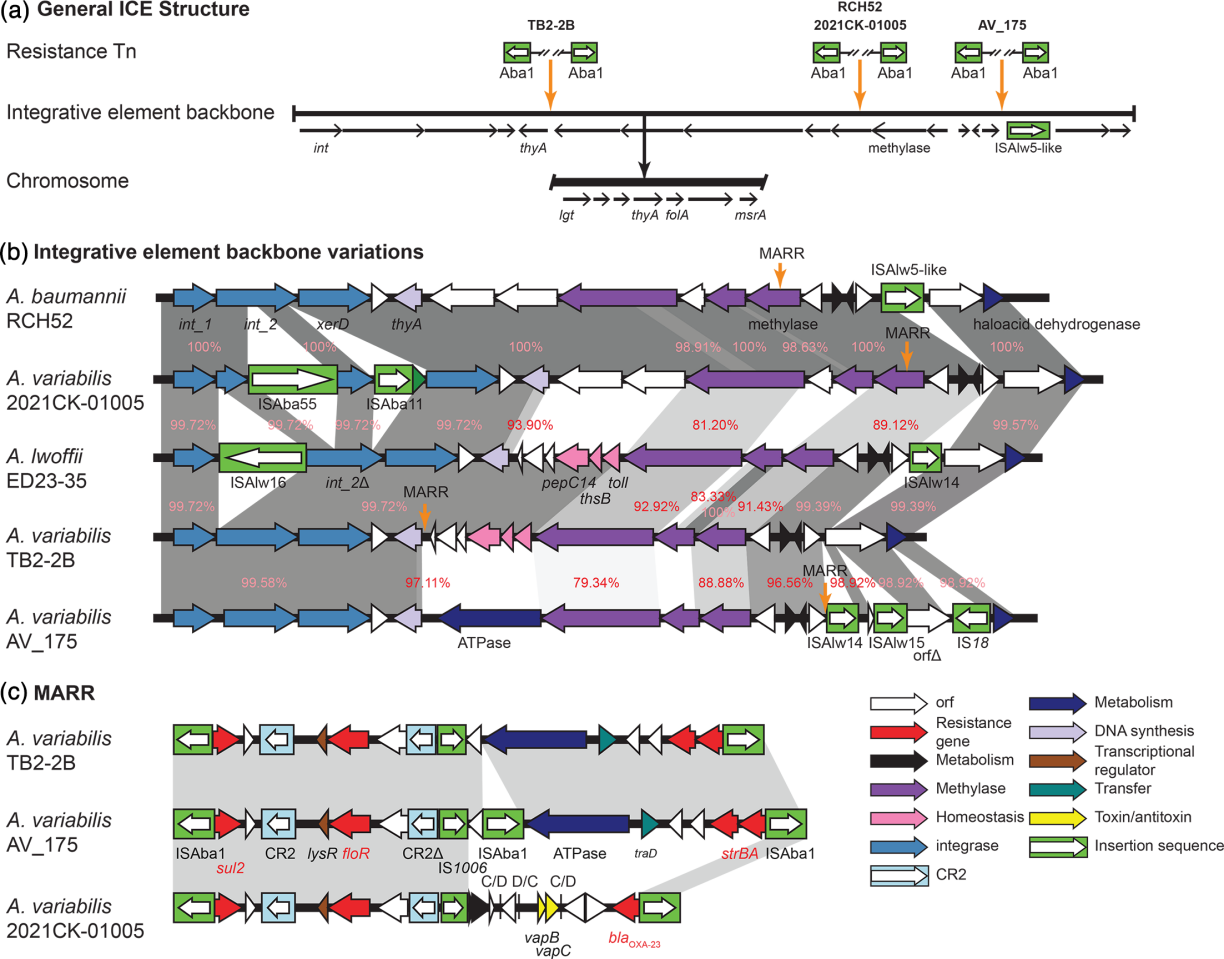
Integrative conjugative element (ICE) in *Acinetobacter variabilis*. (a) General ICE structures, (b) backbone variations in *A. variabilis* and other *Acinetobacter* spp. and (c) variants of ISAba1-bounded multiply antibiotic resistance regions (MARR) in *A. variabilis*. Gene clusters are drawn to scale and genes coloured by their predicted functional groups; arrows show the gene orientation. Open boxes coloured green show insertion sequences with their transposase gene indicated inside the boxes.

We previously characterized a novel ICE in an *A. baumannii* strain (RCH52, recovered in Australia) [43], which contained a 129 kb ISAba1-bounded multiply antibiotic resistance region (MARR), integrated into the chromosomal *thyA* gene (Fig. 5a). This ISAba1-bounded MARR comprised two clusters of resistance genes separated by a large segment of the type 1 IncC plasmid backbone [43]. Our earlier work showed that several resistance genes originating from IncC plasmids had been brought together through an IS*26*-mediated deletion of the original plasmid [43]. This composite unit was then incorporated into an ISAba1-bounded segment containing *bla*_OXA-23_ [43]. Here, we identified several related ICE variants in the chromosomal *thyA* gene in the three complete *A. variabilis* genomes, TB2-2B, AV-175 and 2021CK-01005, with different ISAba1-bounded regions that lacked the IncC plasmid segment. The *thyA* gene was found uninterrupted in NGH-QLD-N1, NIPH 2171 and the five other complete genomes.

The elements identified in TB2-2B, AV-175 and 2021CK-01005 included several differences in the ICE backbones (i.e. additional/missing segments and regions with different identities; Fig. 5b), indicating that these ICE elements belong to a diverse family. The backbone in the TB2-2B genome was also found to be similar to one identified in the genome of *A. lwoffii* strain ED23-35 (Fig. 5b), indicating that they are also widespread across the *Acinetobacter* genus. All three variations identified in *A. variabilis* also included different variants of an ISAba1-bounded MARR integrated at different positions in the ICE backbone (Fig. 5c) compared to the MARR previously described for RCH52 [43]. However, the variant found in *A. variabilis* 2021CK-01005 also contained a *bla*_OXA-23_ carbapenem resistance gene. Genomes containing ICE variants were found in distinct phylogenetic branches and originated from diverse geographical regions, suggesting that these variants were acquired independently.

The *thyA* gene was further assessed in all draft *A. variabilis* genomes and was found interrupted by an ICE backbone in a further five genomes, ULE_I103 (GenBank no. JBIMAH000000000), WU_MDCI_Av06 (GenBank no. JAHPPI000000000), WU_MDCI_Av37 (GenBank no. JAHPQG000000000), AR4517 (GenBank no. JAZHCO000000000) and CTOTU47290 (GenBank no. DAISMA000000000). However, these included a cryptic version of the ICE, as a MARR could not be identified in any of them (not shown in Fig. 5 as structures span multiple contigs). The variations observed in the ICE elements further indicate that they belong to a diverse family that can capture and disseminate important resistance genes (i.e. *bla*_OXA-23_) in *A. variabilis*, and potentially other *Acinetobacter* species.

### Locus for CPS biosynthesis

**Fig. 6.**
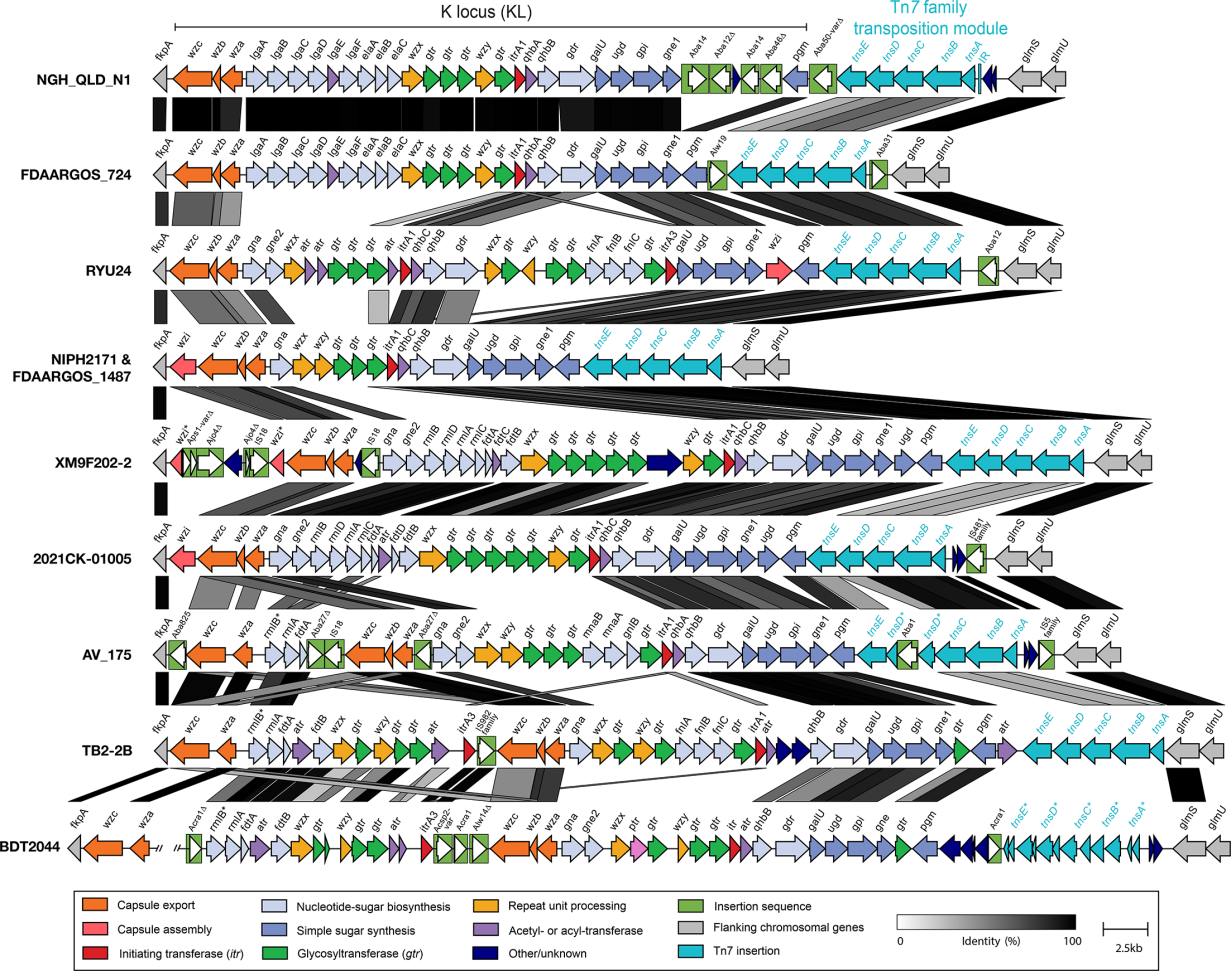
Capsular polysaccharide biosynthesis loci identified in *A. variabilis* complete genomes. Loci are drawn to scale using Clinker, and grey shading indicated sequence identity with scale shown below. Arrows show gene orientation, and genes are coloured by their predicted functional groups with the key shown below. Genes are annotated consistent with the nomenclature scheme for *A. baumannii*. A * suffix indicates gene interruption. The large sequence insertion in BDT2044 was removed and the position of the break is indicated.

The CPS biosynthesis K locus has been studied extensively in *A. baumannii* [25,44, 45] and is located between conserved *fkpA* and *lldP* genes on the chromosome. A region including CPS-associated genes was similarly located immediately upstream of the *fkpA* gene in the chromosome of NGH-QLD-N1 (Fig. 6). The organization of this region is largely analogous to that of * A. baumannii*, with a module of genes (*wza/wzb/wzc*) for CPS export at one end of the locus, and a module of genes (*galU–pgm*) for simple sugar synthesis on the other side. The central region included genes predicting synthesis of 8-epilegionaminic acid (*lgaABCDEF/elaABC*) [46] and bacillosamine (*qhbA/qhbB/gdr*) sugar precursors [25], as well as *wzx* and *wzy* genes, indicating that CPS biosynthesis in *A. variabilis* follows the Wzy-dependent pathway as in *A. baumannii* [25]. The *galU–pgm* module is known to include insertions between *gpi* and *pgm* in *A. baumannii* [25], and this region in NGH-QLD-N1 was found to include IS remnants that flank a gene coding for a hypothetical protein related to type IV pilus proteins.

 For NIPH 2171 and the other complete genomes, a different combination of genes was found at this location. While most carried a unique configuration, some shared modules of genes for the synthesis of complex sugars, which have also been reported in * A. baumannii* previously, e.g. *gna/gne2* for N-acetyl-d-galactosaminuronic acid, *fnlABC* for N-acetyl-l-fucosamine, *rmlBDAC* for l-rhamnose and *qhbA* or *qhbC/qhbB/gdr* for bacillosamine variants [44]. Six of the loci included additional genes between *fkpA* and *wzc* (Fig. 6). Three of these carried a *wzi* gene at this location, though this was interrupted by several IS and IS remnants in XM9F202-2. The three other loci included additional CPS biosynthesis genes in this region, including a second copy of *wza* and *wzc* export genes. However, in the BDT2044 chromosome (NCBI accession number CP094246.1), a large insertion (~250.5 kb) interrupting the locus was identified at this location (Fig. S2). This insertion included two predicted prophage regions and several segments homologous to known *Acinetobacter* plasmids encoding R3-T90 [7]. A large island within this region that includes 13 variant copies of *tet*(X3) conferring tigecycline resistance had been described for BDT2044 previously, and these elements were shown to be potentially mobilizable via the finding of circular intermediates [10].

Unlike *A. baumannii*, *lldP* was not found at the distal end of the locus in any of the *A. variabilis* genomes studied. Instead, several variations of a region, including five genes, were identified downstream between *pgm* and *glmS/glmU*. These five genes encode proteins distantly related to TnsABCDE of Tn*7*, with 39%, 40%, 49%, 38% and 29% amino acid identity, respectively. We also found a 28 bp sequence (TTCTTGTTCGTAATCTACTACTAGTCAC) located 426 bp upstream of the 5′ end of the *tnsA* gene in NGH-QLD-N1 that was absent in other variants. However, we could not detect the other end of the transposon in NGH-QLD-N1 (or any other genome), indicating that it is a remnant of a novel Tn*7*-family transposon. The other end may have been deleted due to an IS-mediated adjacent deletion, given the presence of several IS elements downstream of the *tnsE* gene. In several variants, *tnsA* was located immediately adjacent to the 3′ end of the *glmS* gene, the target site for Tn*7* and related transposons [47]. We also identified this Tn*7*-family transposition module in several draft genomes (including SCsl29, WU_MDCI_Av37, WU_MDCI_Av06, A_187, AR4517, WU_MDCI_Av09, ASP5092 and Ar2053), indicating an early entry into *A. variabilis*.

### Locus for biosynthesis of the LOS outer core

**Fig. 7.**
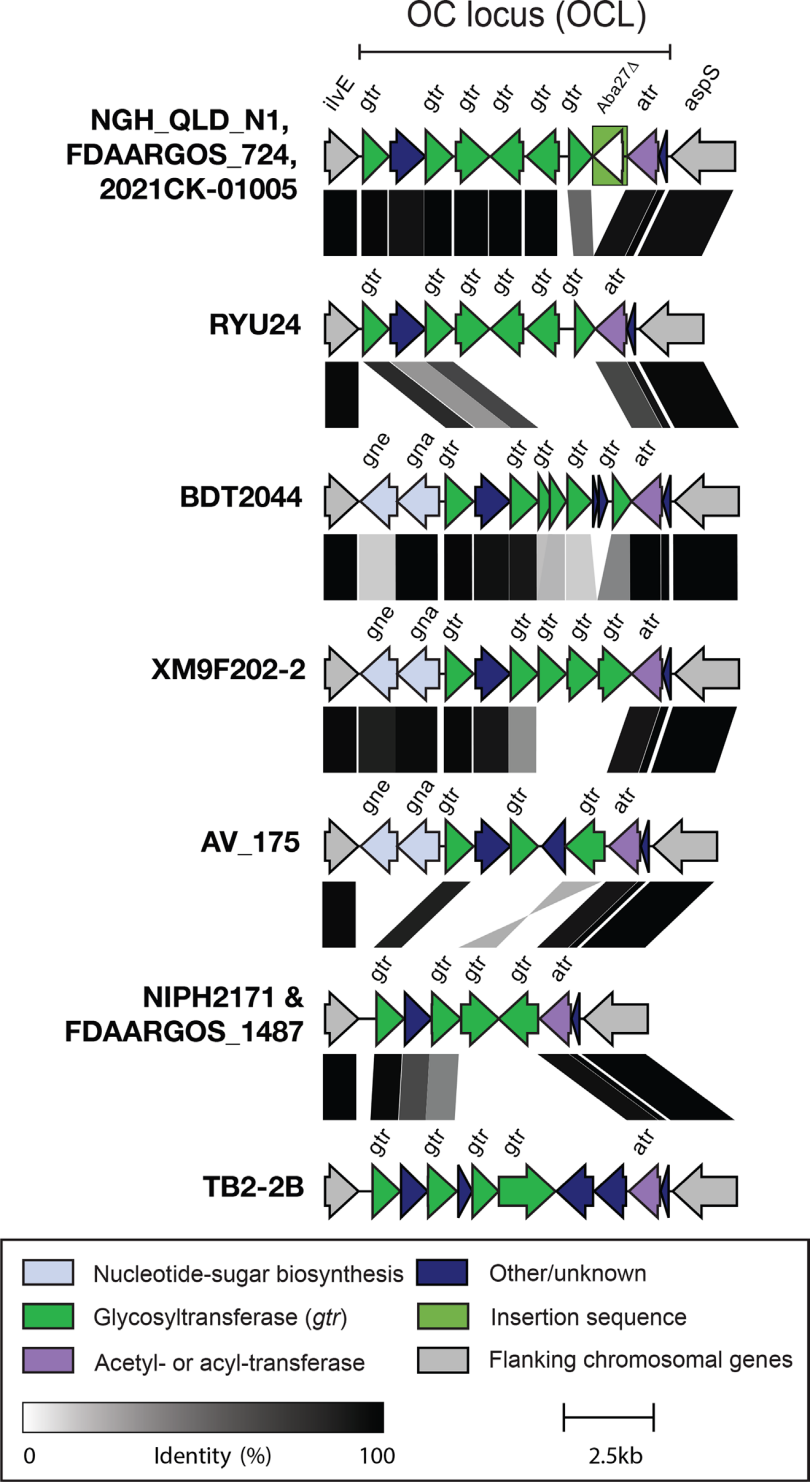
Outer core oligosaccharide biosynthesis loci identified in *A. variabilis* complete genomes. Loci are drawn to scale using Clinker. Grey shading shows sequence identity and scale is below. Genes are coloured by their predicted functional groups with the key below and are annotated consistent with the nomenclature scheme for *A. baumannii*.

In species such as *A. baumannii* that produce LOS, rather than an LPS inclusive of an O-antigen moiety, genes for the outer core (OC) component of the core oligosaccharide of the LOS have been shown to vary substantially [25,48, 49]. In NGH-QLD-N1, NIPH 2171 and the eight complete genomes, a locus including genes predicted to be responsible for OC synthesis was identified between *ilvE* and *aspS* genes. As this is the same location for the OC locus in *A. baumannii* [25,48, 49], this region was also designated as the OC locus in *A. variabilis*. Seven different OC loci were found amongst the ten genomes (Fig. 7), and in general, each was predominately composed of glycosyltransferase genes. NGH-QLD-N1 had the same genetic arrangement as FDAARGOS_724 and 2021CK-01005, whereas NIPH2171 had a different locus type that was also shared with FDAARGOS_1487. The five other genomes carried different locus variations, and all arrangements were different to those previously reported in *A. baumannii* [49].

## Conclusions

This study presents the complete genome sequence of the earliest clinical Australian *A. variabilis* strain, recovered in 2010, and examines its phylogenetic relationship and other genetic features compared with all publicly available *A. variabilis* genomes. We identified major genomic regions, including the CPS and OC biosynthesis loci. In addition, despite harbouring only one resistance determinant, we characterized Tn*6929*, a class III transposon related to the Tn*6022* family, and its variants in the chromosomal *comM* gene of NGH-QLD-N1 and several *A. variabilis* genomes*.* One of these carried *bla*_NDM_ and *aphA6* within an ISAba14-bounded variant region of Tn*125*, while other variants of this transposon were identified on *A. variabilis* plasmids suggesting mobilization of this region and acquisition from diverse bacterial hosts. Multiple ICE variants were also identified in the chromosomal *thyA* gene, differing in backbone structure and resistance gene content from those previously described for * A. baumannii*. These findings highlight Tn*6929* and ICEs as important genomic elements for the capture of resistance genes in * A. variabilis*, particularly in the chromosomal *thyA* and *comM* genes, with potential to spread to other species within the *Acinetobacter* genus. This study serves as a foundational genomic analysis of *A. variabilis* for future studies of this species.

## Supplementary material

10.1099/mgen.0.001643 Uncited Supplementary Material 1.

10.1099/mgen.0.001643 Uncited Table S1.

## Abbreviations

ANIs: average nucleotide identities
ARGs: antibiotic resistance genes
CPS: capsular polysaccharide
ICEs: integrative conjugative elements
LOS: lipooligosaccharide
MARR: multiply antibiotic resistance region
MDR: multidrug-resistant
MLP: maximum-likelihood phylogenetic
NGH: Nambour General Hospital
OC: outer core
SNDs: single-nucleotide differences
TSDs: target site duplications
