# Supplementary material for: Acinetobacter variabilis represents a diverse species with novel regions associated with antibiotic resistance and surface polysaccharides
Source: Microb Genom. 2026 Feb 13;12(2):001643. doi: 10.1099/mgen.0.001643 (PMC12904642; doi:10.1099/mgen.0.001643)
Supplement: Uncited Supplementary Material 1. [file mgen-12-01643-s001.pdf]

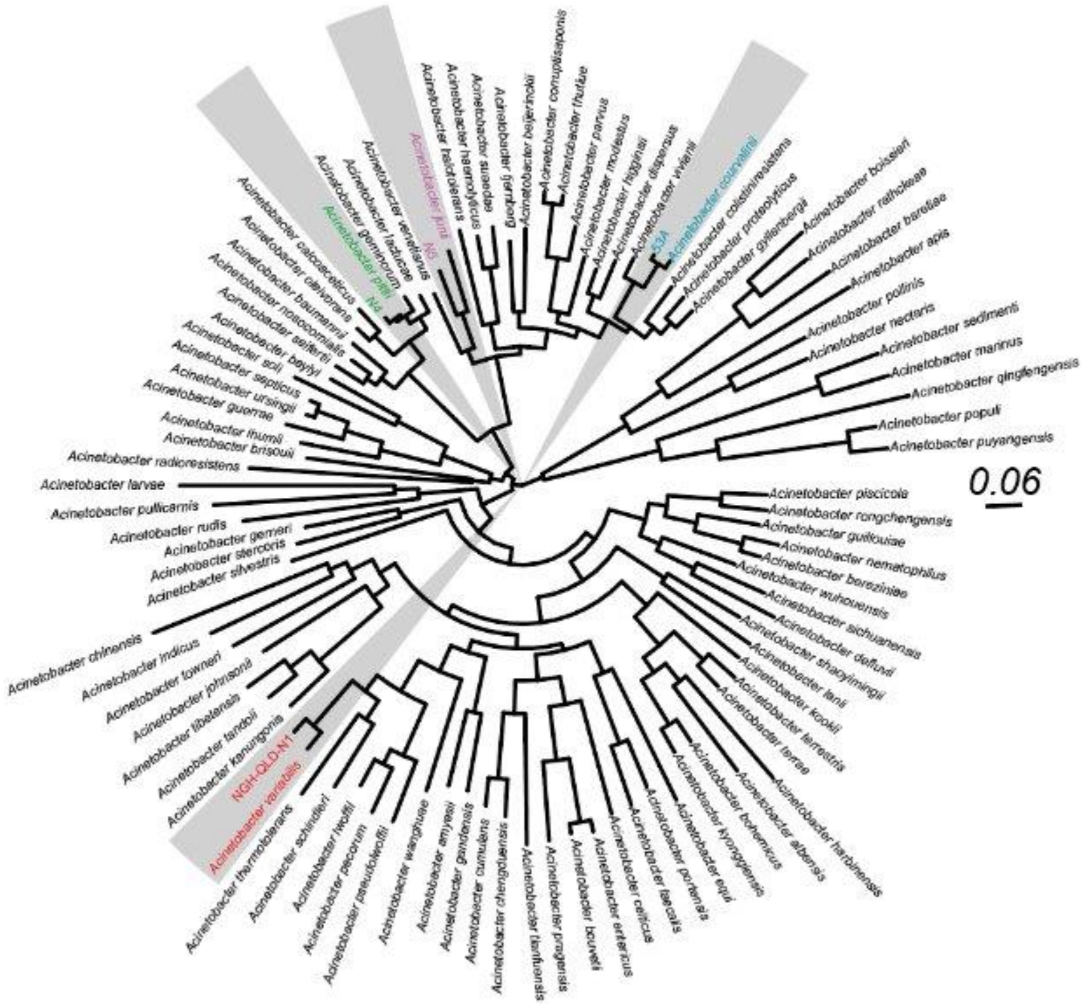

Supplementary Figure S1. A phylogenetic analysis conducted on more than 90 *Acinetobacter* genomes, utilizing one representative genome per species. NGH-QLD-N1 and the reference genome for *A. variablis* are shown red.

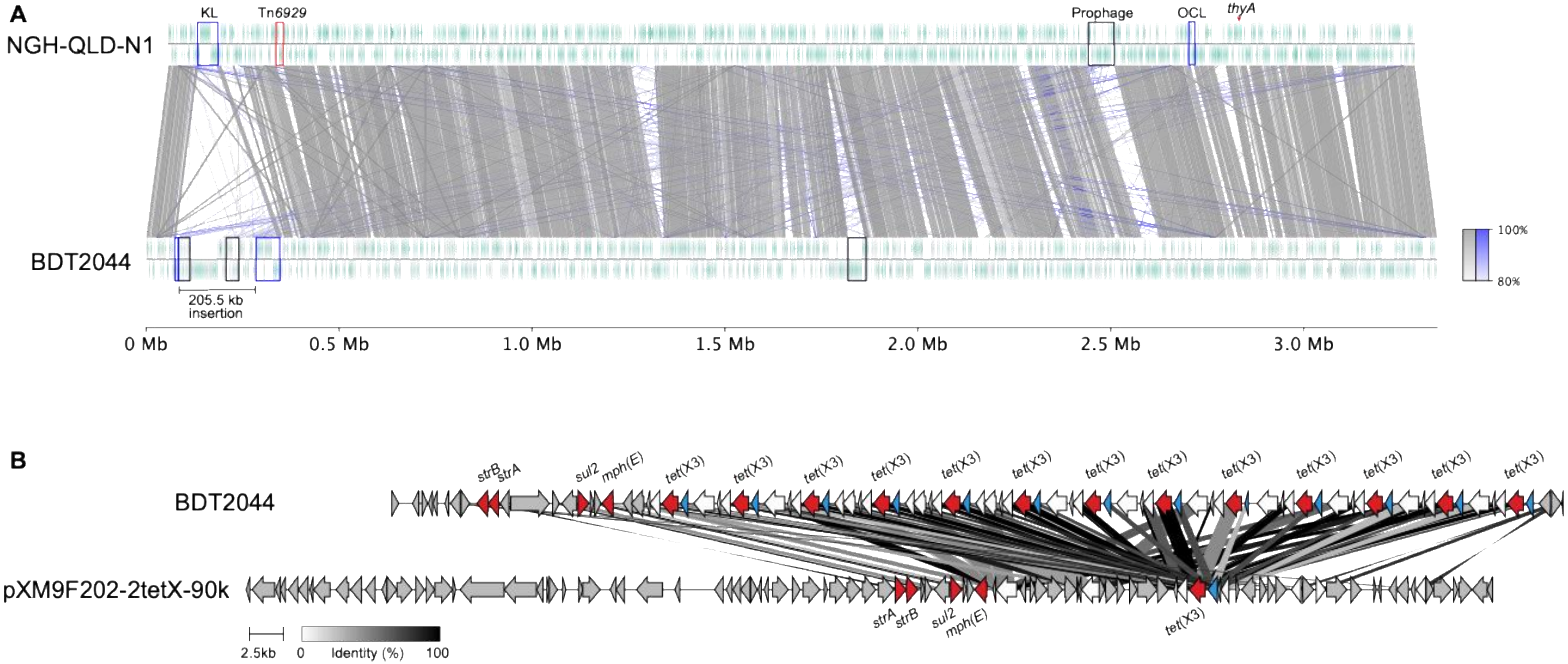

Supplementary Figure S2. Comparison of NGH-QLD-N1 and BDT2044 chromosomes. **A.** Whole chromosome alignment showing region of a large insertion interrupting the K locus in BDT2044. Surface polysaccharide K and OC loci are indicated by blue boxes, prophage regions by black boxes and Tn*6929* and *thyA* regions in red. Shading between chromosomes is % DNA identity with scale shown on the right (blue shading indicates inverted sequence). Figure constructed using pygenomeviz using the blast workflow and then annotated in Adobe Illustrator. **B.** Alignment of the repat region in BDT2044 with pXM9F202-2tetX-90k. Antibiotic resistance determinants are red. XerD type recombinase is blue. Other genes with the tet(X3) segment are white. Shading is % DNA identity with scale shown below. Figure generated using clinker then annotated in Adobe Illustrator.
